# Visceral Leishmaniasis: Advancements in Vaccine Development via Classical and Molecular Approaches

**DOI:** 10.3389/fimmu.2014.00380

**Published:** 2014-08-22

**Authors:** Sumit Joshi, Keerti Rawat, Narendra Kumar Yadav, Vikash Kumar, Mohammad Imran Siddiqi, Anuradha Dube

**Affiliations:** ^1^Division of Parasitology, Central Drug Research Institute, Lucknow, India; ^2^Division of Molecular and Structural Biology, Central Drug Research Institute, Lucknow, India

**Keywords:** visceral leishmaniasis, recombinant vaccines, DNA vaccines, mutant vaccines, synthetic peptide vaccines

## Abstract

Visceral leishmaniasis (VL) or kala-azar, a vector-borne protozoan disease, shows endemicity in larger areas of the tropical, subtropical and the Mediterranean countries. WHO report suggested that an annual incidence of VL is nearly 200,000 to 400,000 cases, resulting in 20,000 to 30,000 deaths per year. Treatment with available anti-leishmanial drugs are not cost effective, with varied efficacies and higher relapse rate, which poses a major challenge to current kala-azar control program in Indian subcontinent. Therefore, a vaccine against VL is imperative and knowing the fact that recovered individuals developed lifelong immunity against re-infection, it is feasible. Vaccine development program, though time taking, has recently gained momentum with the emergence of omic era, i.e., from genomics to immunomics. Classical as well as molecular methodologies have been overtaken with alternative strategies wherein proteomics based knowledge combined with computational techniques (immunoinformatics) speed up the identification and detailed characterization of new antigens for potential vaccine candidates. This may eventually help in the designing of polyvalent synthetic and recombinant chimeric vaccines as an effective intervention measures to control the disease in endemic areas. This review focuses on such newer approaches being utilized for vaccine development against VL.

## Visceral Leishmaniasis: An Unsolved Problem

Visceral leishmaniasis (VL), synonymously known as kala-azar, is caused by obligate intra-macrophage protozoan parasite and is characterized by both diversity and complexity (1). The disease is prevalent in larger areas of tropical, subtropical, and the Mediterranean countries. As per WHO report, nearly 200,000 to 400,000 new cases of VL (with an average duration of several months to more than one year) occur annually with 20,000 to 30,000 deaths per year (http://www.who.int/mediacentre/factsheets/fs375/en/), which is lesser than by malaria among parasitic diseases, although its exact impact has been underestimated as an exact number of cases were never recorded. Ninety percent of the VL cases occur in Bangladesh, Brazil, India, Nepal, and Sudan. In India, 80% VL cases were only from the state of Bihar (2). A sharp ascent in the prevalence of disease is directly related to environmental changes and migration of non-immune people in endemic areas (3). Occurrence of HIV–*Leishmania* co-infection has placed VL as category-1 disease by WHO (4). The arthropod vector – female phlebotomine sandflies, nocturnal, and telmophagous, are responsible for the transmittance of the disease. Two species – *Leishmania donovani donovani* (in East Africa and the Indian subcontinent) and *L. donovani infantum* (in the Mediterranean region of Europe, North Africa, and Latin America) are the main causative organisms for VL (5). The parasite bears two distinct life forms: promastigote, a flagellar form, found in the gut of the vector, which is inoculated into the dermis where it is internalized by dendritic cells and the macrophages and eventually is transformed into an aflagellated amastigote form, which thrives and multiply within the phagolysosomes through a complex parasite–host interaction (6). Current control strategies for VL rely on anti-leishmanial drugs such as pentavalent antimonials, amphotericin B (AmB), miltefosine, paromomycin, etc., but they are far from satisfactory because of their cost, toxicity as well as unpleasant side effects, longer dose schedule with variable efficacies (7). The situation has further worsened with the emergence of resistance against current anti-leishmanial drugs in various regions of endemicity (8). Hence, in the present situation, there is an urgent need to develop an effective vaccine against VL. Although vaccination against VL has received limited attention as compared to cutaneous leishmaniasis (CL), till date, there is no commercial vaccine against any human parasitic disease including leishmaniasis (9). The fact that healing and recovery from the active infection protects individuals from re-infection specifies the possibility of a vaccine against VL (1). An effective vaccine against the disease must rely on the generation of a strong T-cell immunity (10). Both innate (macrophages and neutrophils) as well as adaptive (B-cells, T-cells, and dendritic cells) immune response plays a significant role against *Leishmania* infection where macrophages play the critical role. It has been a consensus for a long time that a Th1 dominant response instead of Th2 promotes IFN-γ production, which activates macrophages to kill parasites via nitric oxide (NO) production, ultimately leading to reduction in parasitic burden (4). The cytokine production and cytotoxic activity by CD8+ T-cells also contribute to the disease outcome in *Leishmania* infection. Initially, CD8+ T-cells were thought to play a role only during re-infection, however, they were also shown to be crucial in controlling the primary infection by skewing the responses toward Th1-type. Effector CD4+ T-cells allow activation of macrophages through various cytokines and are required for optimal host response to infection (11) whereas cytotoxic CD8+ T-cells play a role in parasite clearance with the generation of memory responses (12).

**Figure 1:**
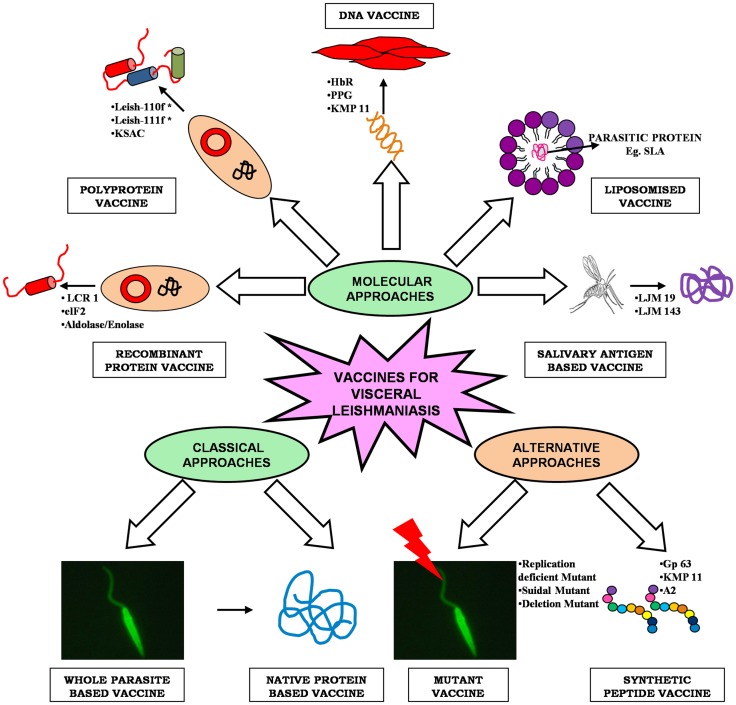
**An overview of different approaches of vaccine development for visceral leishmaniasis**.

As *Leishmania* parasite follows a digenetic life cycle it results in significant antigenic diversity, which ultimately hampered the passage of vaccine development against VL, therefore, the knowledge of such antigenic diversity is of utmost importance (13). Researchers have utilized several approaches for identification of potential antigens, which can be targeted as suitable vaccine candidate (Figure 1). Among them, proteomics attract the most since it addresses several unanswered questions related to microbial pathogens, including its development, evolution, and pathogenicity. Proteomic studies revealed several proteins, which are seen as potential vaccine targets offering varied levels of protection in different animal models. Recent advancement in computational biology further simplifies our knowledge regarding the in-depth study of parasite. T-cell epitope prediction via bioinformatics analysis of protein sequences has been proposed as another alternative for rationale vaccine development (14). The concept that CD8+ T lymphocytes could be important in protection and long-lasting resistance to infection has opened up a new strategy in *Leishmania* vaccine design known as “polytope vaccine” (15). Its major advantages include greater potency, can be controlled better, can be designed to break tolerance, can overcome safety concerns associated with entire organisms or proteins, etc.

## Classical Approaches to *Leishmania* Vaccine Development

### Live/killed whole parasite vaccine

Cutaneous leishmaniasis remained the focus point for earlier attempts for vaccination made in the Middle East due to the fact that people who had their skin lesions healed up were protected lifelong from re-infection. Leishmanization (LZ), the deliberate inoculation of virulent parasite from the exudate of a cutaneous lesion to uninfected individuals, was successfully practiced in Western and South-Western Asia, which offers a strong immunity among individuals through the formation of self-healing lesions (16). As the researchers started culturing promastigote form of parasite in artificial media, the concept of live vaccination came into existence. A number of large-scale vaccination trials were conducted during the 1970s and 1980s in Israel, Iran, and the Soviet Union with a higher success rate. However, standardization and quality control are the major issues associated with live vaccines because parasites used for LZ losses its infectivity due to repeated sub-culturing. Therefore, the focus of vaccine development program was shifted toward killed organisms in the early 90s (17). This concept was abandoned for many years due to the conflicting results obtained in the 40s. However, the vaccination trial conducted in a Brazilian population showed excellent protection with up-regulation of IFN-γ and absence of IL-4, an indicator of long-lasting Th1-type immune response (18, 19). Use of whole killed parasites with or without adjuvant was proposed for both therapeutic as well as for prophylactic purposes (20).

Knowing the fact that deliberate infection of *L. major* to naive people could confer protection against subsequent VL (21) several attempts utilizing this approach was also initiated for the development of vaccine against VL. In this direction, autoclaved *L. major* (ALM) along with BCG was evaluated for its cross protection against VL (Table 1). Dube et al. (22) assessed its protective potential against *L. donovani* challenge in Indian langur monkeys in single as well as triple dose schedules where triple dose schedule was found to be more effective. Immunogenicity of the ALM + BCG vaccine was further enhanced by adsorbing ALM to alum (aluminum hydroxide), which resulted in successful vaccination against *L. donovani* infection in Indian langur monkeys (23). Encouraged with these results Khalil et al. (24) performed a double-blind randomized trial with ALM ± BCG in human subjects against VL in Sudan. None of the evidences showed that ALM + BCG offered significant protective immunity as compared to BCG alone. Here also, the addition of alum improved the immunogenicity of ALM, when administered intradermally (i.d.) at different doses in healthy volunteers from a non-endemic area of Sudan. Results indicated toward the safety of the vaccine mixture, which induced strong delayed type hypersensitivity (DTH) reaction with minimal side effects (25). A similar trial was conducted against canine leishmaniasis in Iran wherein a single injection of alum-ALM + BCG was found to be protective to the tune of 69.3% (26). Killed *Leishmania* can also be given therapeutically in combination with antimonial therapy in order to enhance cure rates and to reduce incidence of relapse (27). However De Luca et al. (28), advocated that autoclaving lowers the immunogenicity of the parasite as it destroys most of the immunogenic proteins. As such Breton et al. (29), applied another approach where they utilized *L. tarentolae*, a non-pathogenic species, to immunize BALB/c mice and found a significant protective immune response after single peritoneal injection against *L. donovani* challenge.

**Table 1:** **Summary of vaccines evaluated against visceral leishmaniasis**.

Vaccine delivery	Antigen	Species used	Challenge with	Host system	Remarks	Reference
**(1) WHOLE PARASITE**						
(a) Killed	ALM ± BCG	*L. major*	*L. donovani*	Indian langur	Triple dose is more effective than single dose	Dube et al. (22)
				Human	Poor efficacy (6%)	Khalil et al. (24)
	Alum-ALM + BCG			Indian langur	Single dose is effective; increased IFN-γ production	Misra et al. (23)
				Human	Protective; induced strong DTH response	Kamil et al. (25)
			*L. infantum*	Dog	Moderate efficacy (69.3%)	Mohebali et al. (26)
(b) Live-attenuated	BT1 deleted parasite	*L. donovani*	*L. donovani*	BALB/c mice	Protective immunity; increased IFN-γ production	Papadopoulou et al. (30)
	SIR2 single allele deletion	*L. infantum*	*L. infantum*		High IFN-γ/IL-10 ratio with increased NO production; protective immunity	Silvestre et al. (31)
	Non-pathogenic strain expressing *L. donovani* A2 antigen	*L. tarentolae*	*L. infantum*		Protective response with high level of IFN-γ production	Mizbani et al. (32)
	Amastigote-specific protein p27	*L. donovani*	*L. donovani*, *L. major*, and *L. braziliensis*		Significant reduction in parasite burden, Th1-type response	Dey et al. (33)
	Suicidal mutant	*L. amazonensis*	*L. donovani*	Hamster	Effective cellular immunity; increased iNOS expression and IFN-γ, IL-12 production	Kumari et al. (34)
	Replication deficient centrin gene	*L. donovani*	*L. donovani* and *L. brazilensis*	BALB/c mice and Hamster	Protective immunity with increased level of IFN-γ, IL-2, and TNF-α producing cells	Selvapandiyan et al. (35)
			*L. infantum*	Beagle dog	High immunogenicity; increased secretion of IFN-γ, TNF-α, IL-12, and decreased production of IL-4	Fiuza et al. (36)
**(2) NATIVE PROTEIN OF PARASITE**						
Parasite fraction	Sonicated antigen+ AlBCG/MISA/MPLA	*L. donovani*	*L. donovani*	Vervet Monkey	Good protection; elicit IFN-γ production	Mutiso et al. (37)
Membrane protein	Dp72 and gp70-2			BALB/c mice	Dp 72 showed 81.1% efficacy; gp70-2 is non-protective	Jaffe et al. (38)
	FML + saponin			Mice	84.4% Protection	Palatnik et al. (39)
				Hamster	Protective	Palatnik et al. (40)
				Mice	Increase in IgG2 and decrease in parasite load by 88%	Santos et al. (41)
			*L. donovani* and *L. chagasi*	Dog	Effective protection; cellular and humoral response	Saraiva et al. (42)
Secretory protein	LiESAp	*L. infantum*	*L. infantum*	Beagle dog	Protective; high level of IFN-γ and low level of IL-4 with increased NO production	Lemesre et al. (43)
					Humoral response with cell-mediated immunity	Bourdoiseau et al. (44)
**(3) RECOMBINANT PROTEIN OF PARASITE**						
Membrane protein	LCR 1	*L. chagasi*	*L. chagasi*	BALB/c mice	Partial protection with increased IFN-γ production but not IL-4, IL-5, and IL-10	Wilson et al. (45)
	HASPB1	*L. donovani*	*L. donovani*	Mice	Protective (70 and 90%); increased IL-12 production by dendritic cells	Stager et al. (12)
	A2			Beagle dog	Partial protection with increased IgG and IFN-γ production; low IL-10 level	Fernandes et al. (46)
Soluble protein	F14	*L. donovani*	*L. donovani*	Golden hamster	Partial protection; increased level of IFN-γ	Bhardwaj et al. (47)
	elF2				Protective (65%); increased level of IFN-γ, IL-12, TNF-α, IgG2, and down-regulation of IL-4, IL-10, TGF-β	Kushawaha et al. (48)
	P45				Protective (85%); increased level of IFN-γ, IL-12, TNF-α, iNOS, and decreased TGF-β, IL-4	Gupta et al. (49)
	PDI				Protective (90%); increased level of IFN-γ, TFN-α, IL-12, and IgG2	Kushawaha et al. (50)
	TPI				Protective (90%); increased level of IFN-γ, TFN-α, IL-12, IgG2, and down-regulation of IL-10, IL-4	Kushawaha et al. (51)
	TPR				Good efficacy (~60%); increased iNOS, IFN-γ, IL-12, TNF-α, and downregualation of IL-4, IL-10, and TGF-β	Khare et al. (52)
	Aldolase and enolase				Increased expression of iNOS, IFN-γ, TNF-α, and IL-12 with down-regulation of TGF-β, IL-4, and IL-10	Gupta et al. (53)
	Ribosomal protein + saponin	*L. infantum*	*L. chagasi*	BALB/c mice	Increased production of IFN-γ, IL-12, and GM-CSF	Chavez-Fumagalli et al. (54)
	Hypothetical amastigote-specific protein		*L. infantum*	BALB/c mice	Protective; increased level of IFN-γ, IL-12, GM-CSF, and down-regulation of IL-4, IL-10	Martins et al. (55)
Secretory protein	Secretory serine protease	*L. donovani*	*L. donovani*	BALB/c mice	Exhibit significant protection with lower parasite burden	Choudhury et al. (56)
	LiESAp-MDP	*L. chagasi*	*L. infantum*	Dog	Efficacy (92%); increased IgG2, NO, and IFN-γ production	Lemesre et al. (57)
**(4) POLYPROTEIN**						
	Q protein	*L. infantum*	*L. infantum*	Dog	Protective (90%); positive DTH response	Molano et al. (58)
				BALB/c mice	Induced significant protection with long-lasting IgG response	Parody et al. (59)
	Leish-111f	*L. major* and *L. braziliensis*	*L. infantum*	Beagle dog	No protection	Gradoni et al. (60)
				Mice and hamster	Decreased parasite load (99.6%); strong Th1 response (increased IFN-γ, IL-2, TNF-α)	Coler et al. (61)
				Dog	Protection	Trigo et al. (62)
	Leish-110f	*L. major*	*L. infantum*	Dog	Protective with increased IFN-γ, TNF-α, and IL-2	Bertholet et al. (63)
	KSAC	*L. infantum* or *L. donovani*	*L. infantum*	C57BL/6 mice	Protective Th1-type response	Goto et al. (64)
**(5) DNA OF PARASITE**						
	A2 DNA	*L. donovani*	*L. donovani*	Mice	Significant protection with increased IFN-γ production	Ghosh et al. (65)
	P36LACK			Mice	Strong Th1-type response (IFN-γ); non-protective	Melby et al. (66)
	ORFF			BALB/c mice	Significant protection (80%) with increased IFN-γ expression	Sukumaran et al. (67)
	KMP-11			Hamster	Mixed Th1/Th2 response; protective with up-regulation of IFN-γ, TNF-α, and IL-12 and down-regulation of IL-10	Basu et al. (68)
				BALB/c mice	Protective; mixed Th1/Th2 response (enhanced IFN-γ and depressed IL-4 production)	Bhaumik et al. (69)
	H2A, H2B, H3, H4, and p36 (LACK)			Dog	Partial protection; elicit type 1 cellular response (IFN-γ)	Saldarriaga et al. (70)
	γGCS			Mice	Protective immunity; production of specific IgG1 and IgG2a antibodies; enhanced granuloma formation	Carter et al. (71)
	UBQ-ORFF			Mice	Protective; higher levels of IL-12 and IFN-γ and the low levels of IL-4 and IL-10	Sharma and Madhubala (72)
	PPG			Hamster	Efficacy about 80% with increased IFN-γ, TNF-α, IL-12, and decreased IL-4, IL-10, TGF-β	Samant et al. (73)
	HbR			BALB/c mice and hamster	Complete protection; increased Th1 response (IFN-γ, TNF-α, IL-12) with down-regulation of IL-4 and IL-10	Guha et al. (74)
	p36 LACK	*L. infantum*	*L. chagasi*	BALB/c mice	Non-protective(IL-10 production); no reduction in parasite load (both liver and spleen)	Marques-da-Silva et al. (75)
	PapLe22			Dog	Downregulate Th2-type response and reduces parasite burden by 50%	Fragaki et al. (76)
	P36 LACK			Mice	Protective immunity; significantly increased IFN-γ and IL-4 with decreased IL-10 production	Gomes et al. (77)
	H2A, H2B, H3, and H4			BALB/c mice	No protection	Carrion et al. (78)
	Purified FML, rNH36, and NH36 DNA	*L. donovani*	*L. chagasi* and *L. mexicana*	BALB/c mice	Significant protection with 88% reduction in parasite load; Th1-type response	Aguilar-Be et al. (79)
	VR1012-NH36		*L. chagasi*	BALB/c mice	Protective (77%); reduction in parasite burden (91%)	Gamboa-Leon et al. (80)
	A2 and NH	*L. chagasi*	*L. chagasi*	BALB/c mice	Protective response (only A2) with increased IFN-γ and decreased IL-4 and IL-10 production	Zanin et al. (81)
**(6) RECOMBINANT PROTEIN + DNA**						
	ORFF (HPB)	*L. donovani*	*L. donovani*	BALB/c mice	Protective; reduction in parasite load (75–80%) with increased IgG2a and IFN-γ production	Tewary et al. (82)
	GP63 as heterologous prime boost (HPB)				Enhanced IFN-γ, IL-12, NO, IgG2a/IgG1 ratio, and reduced IL-4 and IL-10	Mazumder et al. (83)
	Virus expressing LACK antigen (WRp36 or MVAp36)	*L. infantum*	*L. infantum*	BALB/c mice	Protective; significant level of IFN-γ and TNF-α	Dondji et al. (84)
	LACK			Dog	Moderate protection (60%); increased level of IL-4 and IFN-γ	Ramiro et al. (85)
	Type I (*cpb*) and II (*cpa*)			BALB/c mice	Protective; strong Th1 response (higher level of IFN-γ/IL-5 ratio)	Rafati et al. (86)
	CP type I and II		*L. donovani*	Dog	Increased IFN-γ expression and IgG, IgG2 level with strong DTH response	Rafati et al. (87)
**(7) LIPOSOMISED DELIVERY OF PARASITE PROTEINS**						
	Liposomised *L. donovani* antigens	*L. donovani*	*L. donovani*	BALB/c mice	Induced both Th1 and Th2-type responses with high level of IgG2a, IgG2b, and IgG1	Afrin et al. (88)
	pDNA + SLA				Protective; potentiate Th1 response and downregulate Th2 response	Mazumder et al. (89)
	GP63 in stable cationic liposomes				Up-regulation of IFN-γ and down-regulation of IL-4; mixed Th1/Th2-type response	Bhowmick et al. (90)
	BM-DCs pulsed with H1	*L. infantum*	*L. infantum*		Increased level of IFN-γ and IgG2a/IgG1 ratio; decreased level of IL-10	Agallou et al. (91)
**(8) SALIVARY PROTEIN OF VECTOR**						
	*LJM19*	*Lutzomyia longipalpis*	*L. infantum chagasi*	Golden hamster	Protective; high IFN-γ/TGF-β ratio and increased iNOS expression	Gomes et al. (92)
	LJM143 and LJM17		*L. infantum*	Beagle dog	Strong Th1-type response with IFN-γ and IL-12 expression	Collin et al. (93)

*ALM, autoclaved *L*. *major*; BCG, *Mycobacterium bovis* bacillus Calmette Guerin; BT1, biopterin transporter; SIR2, silent information regulatory 2; AlBCG, alum-BCG; MISA, montanide ISA 720; MPLA, monophosphoryl lipid A; dp72, *L. donovani* promastigote antigen of 72 kDa; FML, fucose–mannose ligand; SLA, soluble leishmanial antigens; LiESAp, *L. infantum* excreted-secreted antigen purified; HASPB1, hydrophilic acylated surface protein B1; elF-2, elongation factor-2; PDI, protein disulfide isomerase; TPI, triose phosphate isomerase; MDP, muramyl dipeptide; UBQ-ORFF, ubiquitin open reading frame F; KMP-11, kinetoplastid membrane protein-11; NH, nucleoside hydrolase; LACK, *Leishmania* homolog of receptors for activated C-kinase; γGCS, gamma-glutamyl cysteine synthetase; PPG, proteophosphoglycan; HPB, heterologous prime boost; HbR, hemoglobin receptor; CP, cysteine proteinase; BM-DCs, bone marrow-dendritic cells; TPR, trypanothione reductase*.

Though, whole parasite vaccine (either live/killed or attenuated one) offered vast array of antigens to the host immune system that induced both protective as well as non-protective responses (94), recent advent in our knowledge about the immunobiology of the *Leishmania* infection provided probable explanations for the failure of the first generation vaccines, which further insisted for the development of newer vaccination strategies against VL. A variety of different molecules were identified from parasite based on their abundance, surface localization, T-cell clones, screening of antigen pools/expression libraries with sera of infected animals and humans, which was further evaluated as suitable vaccine candidates leading to the production of a number of experimental vaccines against different forms of leishmaniasis over past few decades (95). In case of VL, extensive vaccination studies have not been possible due to unavailability of an appropriate animal model. Although, golden hamsters and dogs were utilized for studying the immunobiology of *L. donovani* and *L. infantum*, respectively, lack of immunological reagents and assays needed for the characterization of immune responses makes inconclusive study. In such case, a mouse model of VL has been extensively utilized since it exhibit organ-specific pathology in the liver and spleen.

### Protein fractions based vaccine

Selection of suitable vaccine candidates seems to be a difficult task due to the multitude of antigens that has been evaluated with varied success rate depending on their formulation and the type of animal model used (20). Complete protection has not been achieved so far due to the complexity of the parasite, which generates poly-specific response (96). Therefore, different fractions of the parasite in the form of crude preparations were tested as vaccine preparation in order to draw any conclusive results (Table 1). Jaffe et al. (38) demonstrated that mice receiving promastigote-derived membrane protein dp72 yielded a 81.1% reduction in the liver parasitemia as compared with the adjuvant controls, but there has been no further advance on the use of this antigen for the development of vaccines. Another membranous protein, FML, a glycoprotein mixture, of *L. donovani* in combination with saponin was assessed as vaccine in mice, hamster, and dog models of VL and found to be protective (39–42). Lemesre et al. (43) and Bourdoiseau et al. (44) utilized naturally excretory/secretory (ES) antigens of *L. infantum* promastigotes (*Li*ESAp) and found them to be protective in dogs against experimental *L. infantum* infections. Mutiso et al. (37) delivered sonicated antigen of *L. donovani* i.d. with alum-BCG (AlBCG), MISA, or monophosphoryl lipid A (MPLA) in vervet monkeys against homologous challenge and concluded that *L. donovani* sonicated antigen containing MISA is safe and is associated with protective immune response.

A recent meta-analysis of different vaccination trials using these classical approaches had shown the lack of efficacy of these vaccines in clinical trials (97). Also, the efficacy of LZ has not been shown against VL (98). Standardization and quality control are the major problems associated with LZ, which limit its practicality and acceptability (10). Genetic variation and polymorphism in *Leishmania* isolates also deject this approach (99). In case of fraction based vaccines, there are issues related to purity and yield of immunogenic protein. All these lead to explore alternate approaches for generation of better vaccine.

## Molecular Approaches to *Leishmania* Vaccine Development

### Recombinant protein vaccine

With the advancement in recombinant DNA technology, several leishmanial molecules, either species or life cycle stage specific, were extensively studied as a promising vaccine candidate in the form of recombinant proteins. The major advantages associated with these proteins are in terms of purity as well as yield. Numerous proteins were examined against the cutaneous form of diseases, which were later examined against VL when found suitable. LCR1, A2, HASPB1 are the major membrane protein, which was made recombinant and were tested against experimental VL. Wilson et al. (45) identified specific parasite antigens LCR1 from the amastigote stage of the *L. chagasi* that stimulate IFN-γ production and provided partial protection against homologous challenge directing its possible utility in a subunit vaccine. Stager et al. (12) confirmed the role of recombinant hydrophilic acylated surface protein B1 (HASPB1) in protection against *L. donovani* challenge in mice. Fernandes et al. (46) investigated the protective immunity of recombinant A2 protein with saponin against *L. chagasi* infection in dogs where partial protection was noticed with significantly increased IFN-γ and low IL-10 levels (Table 1).

However, several proteins from the soluble fractions of promastigotes stage were also found to be a potent Th1 stimulatory by Kumari et al. (100, 101), which were further developed as recombinant molecules such as protein disulfide isomerase (PDI), triose phosphate isomerase (TPI), elongation factor-2 (elF-2), aldolase, enolase, P45, trypanothione reductase (TPR), etc. Kushawaha et al. (48, 50, 51) studied the immunogenicity of LelF-2, TPI, and PDI of *L. donovani* in PBMCs of cured *Leishmania*-infected patients and hamsters where they found Thl-type cytokine profile (production of IFN-γ, IL-12, and TNF-α but not IL-4 or IL-10) with a remarkable increase in IgG2 and considerable protection. Gupta et al. (49, 53) reported p45, enolase, and aldolase as a potential vaccine candidate with considerable prophylactic efficacy to the tune of 85–90% with an increased mRNA expression of iNOS, IFN-γ, TNF-α, and IL-12 and decrease in TGF-β and IL-4. Vaccination with rLdTPR + BCG provided considerably good prophylactic efficacy (∼60%) against *L. donovani* challenge in hamsters well supported by the increased inducible NO synthase mRNA transcript and Th1-type cytokines IFN-γ, IL-12 and TNF-α and downregulation of IL-4, IL-10 and TGF-β (52). Several other proteins from soluble lysate were also evaluated as recombinant vaccines against VL. For example, recombinant F14 and ribosomal proteins offered partial protection in hamster and BALB/c mice against *L. donovani/L. chagasi* challenges (47, 54). Among the proteins from amastigote stage, recently, a hypothetical *Leishmania* amastigote-specific protein (LiHyp1) was reported to offer protection via IL-12-dependent production of IFN-γ mainly by CD4+ T-cells (55).

Fewer recombinant ES molecules like cysteine proteinases, serine proteases, etc., were also tested as potential vaccine molecule against experimental VL. Lemesre et al. (57) combined ES antigens of *Li*ESAp with muramyl dipeptide (MDP) and found 100% protection in dogs with increased IgG2 and IFN-γ level against homologous challenge. *In vivo* studies of Choudhury et al. (56) in BALB/c mice confirmed serine protease as a potential vaccine candidate.

### Polyprotein vaccine

Due to the genetic polymorphism in the mammalian immune system, a multicomponent vaccine thought to elicit a better protective immune response (64). Therefore, multicomponent or polyprotein preparations such as Q protein, Leish-111f, Leish-110f, KSAC, etc., came into existence that had been demonstrated to afford better protection against experimental VL. Among these, Q protein containing five genetically fused antigenic determinants from Lip2a, Lip2b, H2A, and P0 proteins, was initially assessed along with either BCG or CpG-ODN in mice and dogs (58, 59) against *L. donovani* challenge. Results showed 90% protection with Q protein + BCG in dogs with strong DTH response while Q protein + CpG-ODN motifs were able to induce a long-lasting IgG response in mice. Lately, a phase III trial was conducted in dogs with another potent single polyprotein – Leish-111f, composed of *L. major* homolog of eukaryotic thiol specific antioxidant (TSA), the *L. major* stress-inducible protein-1 (LmSTI-1), and the *L. braziliensis* elongation and initiation factor (LeIF), which was found to be ineffective against *L. infantum* challenge (60). However, when Leish-111f was combined with adjuvant MPLA-stable emulsion (MPL-SE) a significant protection was achieved against experimental *L. infantum* infection in mice and hamsters (61) as well as in dogs (62) with reduction in parasite burden and a cytokine profile indicative of Th1-type immune response. Later on, a new formulation of Leish-111f vaccine – viz Leish-110f was prepared after removal of His-tag, due to the manufacturing and regulatory purposes (102) and was evaluated for its prophylactic potential with different adjuvants [natural (MPL-SE) or synthetic (EM005) toll-like receptor 4 agonists]. This vaccine was also found to be protective, generating good humoral and cellular responses (63). Another defined polyprotein vaccine – KSAC utilizing four proteins, namely, kinetoplastid membrane protein-11 (KMP-11), SMT, A2, and CPB was developed against VL which, along with MPL was found to be immunogenic and offer significant protection against *L. infantum* challenge in mice (64).

Among all these polyprotein vaccines, Leish-110f is under clinical trial in Indian population and the outcome of this vaccination trial is yet to be seen.

### DNA vaccines

Besides proteins, DNA had also been extensively utilized as a means of vaccine delivery, which reformed the area of vaccinology. Here, genes encoding the target proteins are cloned into a mammalian expression vector, which is injected either intradermally or intramuscularly leading to induction of Th1 responses, resulting in strong cytotoxic T-cell immunity. Safety, stability, long-term protection, ease of administration, and cost effectiveness are the major issues associated with this form of vaccine delivery. Several molecules were evaluated using this approach such as A2, PapLe22, P36LACK, ORFF, KMP-11 proteophosphoglycan (PPG), etc., in different animal models with significant level of protection. A2 (65) and ORFF (67) when administered as a DNA vaccine were found to be significantly protective in BALB/c mice against VL, which induced both humoral and cellular immune responses. However, mice immunized with truncated 24-kDa LACK antigen, which, though, generated a robust parasite-specific Th1 immune response (IFN-γ but not IL-4), did not confer any protection in BALB/c mice (66). PapLe22, another protein, was assessed in the golden hamster by Fragaki et al. (76) experienced down-regulation of Th2 response and half reduction of parasitic episodes in blood circulation. The potential of a p36 (LACK) DNA vaccine was evaluated in BALB/c mice against *L. chagasi* wherein no reduction in parasite load (liver and spleen both) was observed, possibly due to IL-10 production (75). On the other hand, Aguilar-Be et al. (79) reported significant protection with the NH36-DNA vaccine against *L. chagasi* in BALB/c mice with 88% reduction in parasite load and with two to fivefold increase in IFN-γ producing CD4+ T-cells confirming Th1-type immune response. Further, Gamboa-Leon et al. (80) used garlic extract with NH36-DNA vaccine, which did not reduce parasite load, but increased survival (100%) with non-specific enhancement of IFN-γ. In an another interesting study, the efficacy of intranasal (i.n.) vaccination with pCIneo-LACK against VL in BALB/c mice was assessed wherein significant reduction in parasite burden was noticed in both liver and spleen along with significantly increased IFN-γ and IL-4 level with decreased IL-10 production (77). Basu et al. (68) and Bhaumik et al. (69) utilized KMP-11 for DNA vaccine in hamsters and BALB/c mice, respectively, where they found significant protection with the mixed Th1/Th2 response (surge of IFN-γ, TNF-α, and IL-12 with extreme down-regulation of IL-10). In another study by Samant et al. (73), vaccination with DNA-encoding N-terminal domain of the PPG gene in golden hamsters yielded 80% protection against the *L. donovani* challenge with generation of Th1 type of immune response. Recently, Guha et al. (74) showed that immunization with hemoglobin receptor (HbR)–DNA induces complete protection against virulent *L. donovani* infection in both BALB/c mice and hamsters with an up-regulation of IFN-γ, IL-12, and TNF-α with concomitant down-regulation of IL-10 and IL-4.

Several enzymes related to protection against oxidative stress were also shown to be better vaccine targets in *Leishmania* as well as in other parasitic diseases. Carter et al. (71) and Sharma and Madhubala (72) vaccinated mice with pVAXγGCS (gamma-glutamyl cysteine synthetase) and UBQ-ORFF, respectively, which resulted in a protective response to increased levels of IL-12 and IFN-γ and the lower levels of IL-4 and IL-10 confirming Th1-type response.

Several workers utilized different antigens in the combinatorial approach in order to enhance the efficacy and protective response of different antigens. Zanin et al. (81) immunized mice with a NH/A2 DNA vaccine resulted in increased IFN-γ, IL-4, and IL-10 levels associated with edema and increased parasite loads. Das et al. (103) very recently have developed a DNA vaccine using conserved proteins from various *Leishmania* species and found to be immunogenic inducing CD4+ and CD8+ T-cell responses in genetically diverse human populations of different endemic regions.

### Heterologous prime boost vaccine

Different researchers utilized another strategy known as heterologous DNA-prime protein-boost (HPB) approach for some VL vaccine antigens such as ORFF, cysteine proteinases, GP63, etc., which have also shown success but are yet to reach the level of clinical trials. Ramiro et al. (85) observed 60% protection in dogs immunized with DNA-LACK prime/rVV-LACK boost against *L. infantum* challenge. Since the immune response in a canine model differs significantly from murine and human hosts, Dondji et al. (84) and Tewary et al. (82) conducted similar studies using the murine intradermal model for VL and found comparable levels of protection. With another combination of cysteine proteinases DNA/protein along with ORFF DNA/protein against experimental VL, Rafati et al. (86, 87) observed that vaccination mainly elicited antigen-specific IgG2a antibodies, suggesting the induction of a Th1 immune response. Very recently, Mazumder et al. (83) evaluated a membrane protein, GP63 in BALB/c mice and found robust cellular and humoral responses correlating with durable protection against *L. donovani* challenge.

### Liposomised delivery of parasite protein

Liposome formulations have been adopted as a drug delivery system against *Leishmania* infection so as to induce an elevated immune response owing to their adjuvant property (104) thus can offer a new approach to the development of VL vaccines wherein it may induce a sustained Th1 immune response. This approach using *L. donovani* promastigote membrane antigens (LAg) encapsulated in positively charged liposomes were found to induce significant protection against experimental VL by Afrin et al. (88). Later, a study conducted by Mazumder et al. (89) showed increase in protective efficacy in animal against homologous challenge with *L. donovani* when vaccinated with both soluble leishmanial antigens (SLA) and non-coding plasmid DNA (pDNA) bearing immunostimulatory sequences (ISS), co-entrapped in cationic liposomes. In another study, using liposomised recombinant membranous protein – GP63 of *L. donovani*, there was a long-term protection against VL in BALB/c mice (90). Recently, vaccination with bone marrow-derived dendritic cells (BM-DCs) – a new delivery system, pulsed with *L. infantum* histone H1 against homologous challenge, Agallou et al. (91) demonstrated antigen-specific splenocyte proliferation with increased IFN-γ and decreased IL-10 production confirming Th1-type immune response.

### Sandfly’s salivary antigen as vaccine

Salivary proteins of vector-sandfly also fetch attraction as a suitable anti-VL vaccine candidates. They received little attention in spite of the fact that salivary proteins from the vector are also delivered to the host during natural transmission of the pathogen and sometimes found immunomodulatory for the host (20). Several salivary proteins of *Phlebotomus* spp. and *Lutzomyia* spp. such as PpSP15, maxadilan, LJM17, LJM19, and LJM143 have been reported as potent immunogens inducing lymphocytic infiltration with up-regulation of IFN-γ and IL-12 (92, 93). Although, these proteins conferred protection against CL (105, 106) they were also assessed for their immunogenicity as well as a protective response against VL. LJM19, an 11 kDa protein, was found to be protective with higher expression of IFN-γ and a strong DTH response in a hamster model (92). Similarly, immunization with other two salivary proteins – LJL143 and LJM17 generated strong Th1 responses in dogs with distinct cellular infiltration of CD3 + lymphocytes and macrophages (93). Therefore, these proteins may further be explored in conjunction with potent parasite proteins for vaccination studies.

Despite these different approaches offer a variable degree of efficacy, several problems still hampers its feasibility due to variations in immunogenicity and due to genetic variation in host as well as in pathogen (99). Therefore, despite of numerous recombinant proteins that have been suggested as potential vaccine candidates, to date barely few have reached to clinical trials (107). Similarly, DNA vaccine faces problems in terms of demonstration of safety and efficacy in humans in clinical trial (99).

## Newer Alternative Strategies for Developing Anti-Leishmanial Vaccine

### Live mutant vaccine

Attenuation of virulent *Leishmania* parasites through defined genetic alteration is a new area in vaccine research since the perception of vaccination suggests that the more similar a vaccine is to the natural disease, better is the generation of protective immune response (108). Poor long-term immunity is the major issue with various recombinant vaccines tested so far while whole cell killed vaccines showed variable efficacy. Consequently, live-attenuated vaccine attracts the immunologists, since, it offers a complete milieu of antigens to the antigen presenting cells (APCs), therefore, providing an optimal polarization of CD4+ T-cells, resulting in better immune response (109). Also, they assure persistence of antigen that may allow the generation of antigen-specific effector and memory cells, which react immediately following infection (110). However, till date, only limited attenuated strains have been tested with various outcomes. Earlier construct generated by gene replacement was *dhfr-ts* – and *lpg2* – mutants of *L. major* and *L. mexicana* (111) that were excluded as future *Leishmania* vaccines due to some inherent problem, but still they did open the door for live-attenuated vaccine against VL. Papadopoulou et al. (30) inactivated the *L. donovani* biopterin transporter BT1 by gene disruption that elicits protective immunity in mice against a *L. donovani* challenge (Table 1). However, Silvestre et al. (31) inactivated one allele of SIR2 in *L. infantum*, which elicits complete protection in BALB/c mice with generation of specific anti-*Leishmania* IgG antibody subclasses and increased IFN-γ/IL-10 ratio indicating both type 1 and type 2 responses. Mizbani et al. (32) stably expressed the *L. donovani* A2 antigen in *L. tarentolae* to check its protective efficacy in BALB/c mice against *L. infantum*. Results showed increased production of IFN-γ followed by reducing levels of IL-5 when administered intraperitoneally indicates potential Th1 immune response. In contrast, intravenous injection elicited a Th2-type response, characterized by higher levels of IL-5 and high humoral immune response, resulting in a less efficient protection.

Recent investigations have established that tumor cells treated *in vitro* by photodynamic therapy (PDT) can be used for generating potent vaccines against cancers of the same origin. *Leishmania*, naturally residing in the phagolysosomes of macrophages, is a suitable carrier for vaccine delivery. Genetic complementation of *Leishmania* to partially rectify their defective heme-biosynthesis renders them inducible with delta-aminolevulinate to develop porphyria for selective photolysis, leaving infected host cells unscathed. Delivery of released “vaccines” to APCs is thus expected to enhance immune response, while their self-destruction presents added advantages of safety. Such suicidal *L. amazonensis* was found to confer immunoprophylaxis and immunotherapy on hamsters against *L. donovani* (34).

Centrin, a growth regulated gene was deleted from the amastigote stage of the *L. donovani* parasite and was subjected to evaluation of its prophylactic potential (112). The *LdCen*^−/−^ parasite was found to be safe and protective in mice and hamsters against virulent challenge (35) and is under exploration for further development as potential vaccine against VL. Fiuza et al. (36) presented an immunogenicity profile of LdCen^−/−^ in dogs and showed increased antibody production and amplified lymphoproliferative response. Further, LdCen^−/−^ vaccinated dogs showed higher frequencies of activated CD4+ and CD8+ T-cells, IFN-γ production by CD8+ T-cells, increased secretion of TNF-α and IL-12/IL-23p40 and decreased secretion of IL-4. Very recently, Dey et al. (33) have demonstrated another knock out – Ldp27 (−/−) parasites to be safe and can provide protective immunity against both homologous and heterologous challenge with stimulation of both Th1-type CD4+ and CD8+ T-cells. Since, effector T-cell population requires continuous stimulation for excellent protection; it can be well accomplished through live-attenuated vaccines. Although, there are certain issues associated with these vaccines such as probable reversal to virulence, reactivation in immune compromised individuals, manufacturing considerations, restraint to their usage in clinical studies due to the presence of antibiotic resistance genes used as selective markers during the steps of gene deletion, etc., the two-step approach, i.e., gene deletion with parasite selection and excision of the antibiotic gene cassette offers a promising way toward the generation of a safe live-attenuated vaccine. Thus, all these approaches pave the way for the development of newer generation of vaccine, which would rather be safer, provide long-lasting immunity and meet both scientific as well as regulatory standards.

### Synthetic peptide vaccine

Recent developments in blending of bioinformatics with vaccinology has revolutionized and expedited this area. Sequencing of large number of pathogen genome and increase in nucleotide and protein sequence databases accelerate the pace of vaccine development program. Although, killed or attenuated parasites are utilized for most of the existing vaccines, protective immune response is more often triggered by small amino acid sequence (peptides). More recent bioinformatic approaches utilizes number of algorithms for predicting epitopes, HLA-binding, transporter of antigen processing (TAP) affinity, proteasomal cleavage, etc., in order to explore the use of peptide epitopes with the highest probability of inducing protective immune responses. Generation of synthetic polyvalent peptide vaccine requires better understanding of T- and B-cell epitopes in the microorganism’s proteins and their interaction with major histocompatibility (MHC) or HLA complexes. The basis of using such peptide epitopes arises from the screening of hundreds of overlapping synthetic peptides, which revealed that only a small number of regions in a protein are immunogenic and capable of provoking humoral as well as cellular immune responses. Synthetic peptide vaccines offer several advantages over other vaccine types like absence of any potentially infectious material, ability to include multiple epitopes, minimization of the amount and complexity of an antigen, economical scale up and decreased chance of stimulating a response against self-antigens.

T-cell epitopes are presented on APC surface where they interact with MHC molecules in order to induce immune response. They can be categorized as conformational or linear, depending on their structure and integration with the paratope. One of the key issues in T-cell epitope prediction is the prediction of MHC binding as it is considered a pre-requisite for T-cell recognition. All T-cell epitopes are good MHC binders, but not all good MHC binders are T-cell epitopes. For epitope prediction, generally two methods are adopted, first, sequence based that analyze protein sequences and second, structure based method using three-dimensional protein structures. Whether the predicted epitopes interact with paratope or not can also be assessed by using computational tools, which determines protein–protein interactions that helps in designing novel vaccines. Several strategies such as genomic databases, evolutionary relationships, three-dimensional structure of proteins, presence of specific protein domains, primary structure of proteins, etc., have been applied to knowhow novel interacting partners in order to validate the presumed interactions. Due to the availability of epitope mapping and binding prediction algorithms, several workers have applied different bioinformatic approaches to design synthetic peptide vaccines against several parasitic diseases. In case of malaria, there have been nine clinical trials from 2000 to 2009 utilizing synthetic peptide vaccines, which target the pre-erythrocytic and erythrocytic stages of the *Plasmodium falciparum*, with encouraging results (113). Similarly, this approach has also been utilized in other parasitic diseases such as *Toxoplasma* (114), *Trypanosoma* (115), etc.

In case of *Leishmania*, several proteins like glycoprotein 63 (GP63), KMP-11, amastigote virulence factor (A2), lipophosphoglycan (LPG), cysteine proteinase, etc., both from promastigote as well as amastigote form were screened for determination of potential antigenic peptides for generation of peptide vaccine (Table 2).

**Table 2:** **Summary of peptide vaccines evaluated against leishmaniasis**.

Protein(s)	Spp. used	Epitopes (no. of amino acid residues)	Prediction tool(s) utilized	Challenge with	Dose and route	Host system	Immune response	Reference
GP63	*L. major*	PT 1–4; PT 6–8 (12–16 residues)	Predictive algorithm	*L. major*	100 μg (each) + 8% poloxamer 407; SC	BALB/c mice	Proliferation of CD4^+^ Th1 sub-set cells PT3 showed immunoprotection	Jardim et al. (116)
		24 Partially overlapping peptides (12–35 residues)	AMPHI algorithm	*L. major*	100 μg + 100 μg *C. parvum*/entrapped within liposomes; SC/IV	CBA and BALB/c mice	Induction of T-cell response; classical DTH reactivity and secretion of IL-2 and IFN-γ p146-171 and p467-482 induces significant host-resistance	Yang et al. (117)
		P154 and P467 (16 residues)	AMPHI algorithm	*L. major*	50 μg; IP or SC	CBA mice	ThI type cytokine responses Secretion of IL-2, IFN-γ, and GM-CSF	Frankenburg et al. (118)
		PT3 (16 residues)	Predictive algorithm	*L. major*	100 μg + 8% poloxamer 407; SC	BALB/c mice	Long-lasting protection	Spitzer et al. (119)
		MHC class II – restricted peptides (AAR, AAP, ASR) (15 residues)	SYFPETHI		100 μg emulsified in 1:1 dilution with IFA; SC	FVB/N-DR1 transgenic mice	High levels of Th1-type immune response and significant level of IFN-γ	Rezvan (120)
	*L. mexicana/L. major*	HLA-A2 peptides (9 residues)	SYFPETHI		100 μg + 140μg HAP-B (helper peptide) + 50 μl IFA; SC	HHDII and BALB/c mice	Induction of CTL responses Up-regulation of IFN-γ	Rezvan et al. (121)
	*L. donovani*	P1–P4 (9–18 residues)	EpiMatrix		100 μg of each peptide	Human PBMCs	Moderate increase in IFN-γ	Elfaki et al. (122)
KMP-11	*L. donovani*	84 Overlapping peptides (9 residues)	SYFPEITHI	*L. donovani*	44 μg/ml (each)	CD8^+^ T-cells from human PBMCs	Trigger interferon-γ secretion by CD8^+^ T-cells	Basu et al. (123)
A2	*L. donovani*	MHC class I binding peptide (CD8) and B-cell epitope (B-1) 2 MHC class II binding epitopes (CD4-1 and CD4-2; 17 residues each)	BIMAS and ProtScale	*L. chagasi*	CFSE (20 μm) cells pulsed for 30 min at 37°C + A2-specific peptide + CFSE (1 μm each), and injected at 4 × 10^7^ cells/mouse; IV	BALB/c and C57BL/6 mice	Induction of both IFN-γ secreting CD4^+^ T and CD8^+^ T-cells as well as cytolytic CD8^+^ T-cells	Resende et al. (124)
CPB, CPC LmsTI-1, TSA, LeIF, and LPG-3	*L. major*	18 HLA-A*0201 restricted peptides (9 residues)	SYFPEITHI, BIMAS, EpiJen, Rankpep, nHLApred, NetCTL, and Multipred	*L. major*	10 μg/ml	Human PBMCs	Induces CD8^+^ T-cell response	Seyed et al. (125)

*GP63, glycoprotein 63; KMP-11, kinetoplastid membrane protein-11; A2, amastigote virulence factor; CPB, type I cysteine proteinase; CPC, type III cysteine peptidase; LmsTI-1, *L. major* stress-inducible protein; TSA, thiol specific antioxidant; LeIF, elongation initiation factor-2 alpha subunit; LPG-3, lipophosphoglycan biosynthetic protein; CPA, cysteine peptidase A*.

#### Glycoprotein 63

GP63 also known as leishmanolysin, is the most widely studied protein, which is highly conserved among all leishmanial species. This zinc metalloprotease is expressed well both in promastigote as well as in amastigote form and implicated in a number of mechanisms related to parasite virulence. Also, proteinase activity of leishmanolysin results in increased resistance to complement-mediated lysis. All these make it an attractive vaccine candidate. As early as in 1990, Jardim et al. (116) utilized primary structure of GP63 to delineate the structures of 7 T-cell epitopes (12–16 residues), which stimulate the proliferation of CD4+ cells. One of these synthetic antigens (with adjuvant) showed proliferation of the Thl subset when inoculated subcutaneously and provided immunoprotection against two species of *Leishmania* parasites. Eleven T-cell epitopes out of 24 partially overlapping peptides (12–35 residues) of GP63 of *L. major* have been identified and their prophylactic efficacy was assessed in CBA and BALB/c mice against *L. major* challenge. These epitopes induce a T-cell response suggesting GP63 as a dominant T-cell inducer *in vivo*. There is a clear segregation of the antigenicity and the immunogenicity of the peptides; only 3 of the 11 stimulatory peptides were able to induce a T-cell response as well as being recognized by T-cells from recovered mice. Frankenburg et al. (118) also tested two peptides representing predicted T-cell epitopes of GP63 of *L. major* in vaccines tested in murine model of CL. Either subcutaneous (s.c.) or intraperitoneal (i.p.) immunization in saline with a peptide representing GP63 amino acids 467–482 (p467) significantly protected CBA mice against the development of severe cutaneous lesions only when the peptide was intrinsically adjuvanted by covalently adding a lauryl cysteine moiety (LC-p467) to its amino terminus during synthesis. A single synthetic T-cell epitope (PT3) was obtained from the histidine zinc-binding region of GP63 and was utilized in a vaccine trial using two virulent strains of *L. major* by Spitzer et al. (119). A single s.c. injection of PT3 with poloxamer 407 protected BALB/c mice for 10 months. Protection was similar for both strains, which manifest different disease sequelae. Elfaki et al. (122) used EpiMatrix algorithm to select putative T-cell epitopes of *L. donovani* GP63 in order to assess their immunogenicity *in vitro*. They found significant reduction in IL-10 level in all individual peptides as compared with unstimulated controls. Also, pooled peptides showed moderate increase in IFN-γ level in some volunteers while individual peptides did not show significant difference from negative controls. Similarly, four HLA-A2 peptides of *L. mexicana/major* GP63 were predicted by SYFPETHI and tested in HHD II mice. Results revealed immunogenicity for three of four peptides predicted for HLA-A2 with induction of CTL responses detected by standard 4-h cytotoxicity assay and significant up-regulation of IFN-γ. When HHDII mice were injected i.m with *L. mexicana* GP63 cDNA and splenocytes were restimulated with blasts loaded with the immunogenic peptides, two of the peptides induced significant level of IFN-γ detected by ELISA (121). Recently, three MHC class II – restricted peptides (AAR, AAP, and ASR) from *L. major* GP63 protein were predicted by SYFPEITHI and tested in FVB/N-DR1 transgenic mice. AAR produced high levels of Th1-type immune response as well as IFN-γ (120).

#### Kinetoplastid membrane protein-11

An 11 kDa highly conserved protein exclusively present in parasite cell membrane, differentially expressed more in amastigotes than in promastigotes, which further increases during metacyclogenesis, plays crucial role in host–parasite interaction (126). Basu et al. (123) scanned the entire sequence of KMP-11 of *Leishmania* with overlapping nonapeptides to decipher the role of CD8+ T-cells in defense against infection and in the cure of the disease. Thirty peptides that specifically trigger interferon-γ secretion by human CD8+ T-cells were identified. Four T-cell lines with specificities for different peptides recognize *Leishmania*-infected autologous macrophages, which prove that KMP-11 is processed and presented via the MHC class I pathway of infected cells.

#### A2 protein

It is a member of amastigote stage-specific protein family, identified in *L. donovani*, required for the survival of amastigotes in visceral organs of mammalian host (127). It consists of multiple copies of a decameric amino acid repeat thus ranges from 45 to 100 kDa inducing a strong Th1 immune response thus conferring partial protection against natural infection. Resende et al. (124) predicted hydrophilic, class I and II MHC-binding synthetic peptides recognized by A2-specific antibodies, CD8+ T and CD4+ T-cells, respectively. Immunization of BALB/c mice with adenovirus expressing A2 (AdA2) resulted in low antibody response, contrasting with high levels of IFN-γ producing CD4+ T and CD8+ T-cells specific for A2. Further, A2-specific CD8+ T-cells from immunized mice were capable of lysing sensitized target cells *in vivo*. They further demonstrated an association of A2-specific T-cell responses and reduced parasitism in both liver and spleen from mice immunized with AdA2 and challenged with *L. (L.) chagasi*. Six *L. major* antigens (CPB, CPC, LmsTI-1, TSA, LeIF, and LPG-3) were screened for potential CD8+ T-cell activating 9-mer epitopes presented by HLA-A*0201. Specific response to LmsTI-1 and LPG-3-related peptides presented in HLA-A*0201 was demonstrated (125). Recently, Agallou et al. (128) analyzed eight peptides from four known antigenic *L. infantum* proteins, i.e., cysteine peptidase A (CPA), histone H1, KMP-11, and *Leishmania* eukaryotic initiation factor (LeIF) for their immunogenicity in BALB/c mice where they found that CPA_p2, CPA_p3, LeIF_p3, and LeIF_p6 induced IFN-γ producing CD4+ T-cells indicating a Th1-type response. In addition, CPA_p2, CPA_p3, and H1_p1 also induced CD8+ T-cells.

## Concluding Remarks

For effective intervention measures to control VL in endemic areas, it is imperative to design a vaccine, which is the most economical way of controlling infectious diseases. An ideal vaccine involves suitable vaccine candidates, ought to offer long-lasting immunity, which is the prime pre-requisite for evaluating the efficacy of a vaccine. Although researchers utilize different approaches for designing vaccines against VL, they still face several challenges either due to heterogeneity of the human population or due to unusual host evasive mechanisms of parasite. The key step in vaccine designing is the identification of most appropriate vaccine candidate, which is found to be a time consuming and labor-intensive task. Therefore, efforts were made for rationale and faster identification of potential antigens. With the emergence of immunoinformatics, peptide-based vaccines attract the most due its several merits. These vaccines should include promiscuous T-cell epitopes derived from the potential Th1 stimulatory proteins of *L. donovani*, which expands host protective immune responses.

## Conflict of Interest Statement

The authors declare that the research was conducted in the absence of any commercial or financial relationships that could be construed as a potential conflict of interest.
